# Effect of Heat Treatment on Structural, Magnetic and Electrical Properties of La_2_FeMnO_6_

**DOI:** 10.3390/ma14247501

**Published:** 2021-12-07

**Authors:** Djoko Triyono, Y Yunida, Rifqi Almusawi Rafsanjani

**Affiliations:** Department of Physics, Faculty of Mathematics and Natural Science (FMIPA), Universitas Indonesia Depok, Depok 16424, Indonesia; yunida128@gmail.com (Y.Y.); rifqialmusawi@gmail.com (R.A.R.)

**Keywords:** La_2_FeMnO_6_, calcination, sintering, crystallite size, magnetisation, conduction

## Abstract

In this study, the effect of heat treatment on the structural, magnetic and electrical properties of La_2_FeMnO_6_ prepared via the sol–gel and sintering method were investigated. The heat-treatment conditions, i.e., the calcination temperature (1023 K and 1173 K), sintering temperature and time (1273 K for 1 and 3 h) were carried out. X-ray diffraction (XRD) revealed orthorhombic *pnma* (62) symmetry without any impurity phase for all samples. X-ray photoelectron spectroscopy confirmed the presence of Fe^2+^–Fe^3+^–Fe^4+^ and Mn^3+^–Mn^4+^ mixed states, and lanthanum and oxygen vacancies resulting in various magnetic exchange interactions. Furthermore, the magnetisation hysteresis showed enhanced hysteresis loops accompanied by an increase in magnetisation parameters with calcination temperature. The Raman phonon parameters induced a redshift in the phonon modes, alongside an increase in the intensity and compression of the linewidth, reflecting a decrease in lattice distortion, which was confirmed by XRD. The temperature-dependent conductivity showed that the conduction mechanism is dominated by p-type polaron hopping, and the lowest activation energy was approximately 0.237 ± 0.003 eV for the minimum heat-treatment conditions. These results show that varying heat-treatment conditions can significantly affect the structural, magnetic and electrical properties of the La_2_FeMnO_6_ system.

## 1. Introduction

Double perovskite oxides, $A_{2}{\mathrm{BB}\prime O}_{6}$, in which $B^{\prime }$ and $B^{\prime \prime }\;$are occupied by different transition metals, have attracted significant attention and have been intensively studied of late [1,2,3,4,5]. Starting from half-metal ferrimagnetic Sr_2_FeMoO_6_ (SrFeO_3_–SrMoO_3_) with a fair Curie temperature (approximately 415 K) and showing potential in spintronic applications, the combination of other perovskites has attracted remarkable research interest [2]. Moreover, the synthesis method affects the structural symmetry, magnetic, optical and electrical transport properties of the synthesised material [3,4].

Among the double perovskites, La_2_FeMnO_6_ has gained much interest owing to its structural, magnetic and electrical properties, which are strongly influenced by the preparation conditions and methods [3,4,5,6,7]. Palakkal et al. [3] stated that La_2_FeMnO_6_ synthesised via citrate–nitrate gel combustion has an orthorhombic phase with the Pbnm space group, a spin-glass state and various super-exchange interactions due to the mixed Fe^2+^−Fe^3+^ and Mn^3+^−Mn^4+^ within the system. Dhilip et al. [4] reported that La_2_FeMnO_6_ prepared by high-temperature solid-state reactions possesses a cubic symmetry and strong competing antiferromagnetic–ferrimagnetic (AFM–FIM) interactions. Filho et al. [5] investigated La_2_FeMnO_6_ nanoparticles synthesised via ionic coordination reactions, which exhibited monoclinic P_21/n_ symmetry at room temperature, spin-glass behaviour with the transition temperature of approximately 98.9 K and an exchange bias effect below 20 K. Yang et al. [6] reported the occurrence of spin without strain coupling in the paramagnetic state–spin cluster state transition near 270 K for La_2_FeMnO_6_ synthesised via conventional solid-state reactions. Li et al. [7] reported that La_2_FeMnO_6_ prepared using the citrate-gel method preserves the orthorhombic Pnma symmetry up to 87.8 GPa accompanied by the first-order phase transition in the range of 28–45 GPa.

Most studies have been limited to investigating the low-temperature properties and certain heat preparation conditions. However, there is a need to thoroughly understand the effect of heating conditions on La_2_FeMnO_6_ to optimise the material properties, which are crucial for fundamental scientific and electrochemical applications. In this study, the effect of heat treatment on the structural, magnetic and electrical properties of La_2_FeMnO_6_ were investigated. The calcination temperature was varied from 1023 K to 1173 K to evaluate its effect on the structural and magnetic properties. The sintering condition was varied between 1 and 3 h at 1273 K to investigate its effect on the structural and electrical properties of La_2_FeMnO_6_. The results of these investigations are discussed below.

## 2. Materials and Methods

A double perovskite La_2_FeMnO_6_ sample was prepared using the sol–gel and sintering method under the following procedure [7]. Analytical-grade lanthanum (II) oxide (La_2_O_3_, 99.9%), iron (II) nitrate nonahydrate (Fe(NO_3_)_3_·9H_2_O, 99.99%) and manganese (II) nitrate tetrahydrate (Mn(NO3)_2_·4H_2_O, 99.98%) were obtained from Merck. To obtain La_2_FeMnO_6_ powder, the precursors were dissolved, based on stoichiometry calculations, into a 0.5 molar concentration of citric acid monohydrate. The solution was continuously stirred at 450 rpm and heated at 120 °C due to the evaporation of the reagents and a gel was formed afterwards. The gel was dried and calcined to obtain the powdered La_2_FeMnO_6_ sample. The optimum calcination conditions were varied from 1023 K to 1173 K. The crystal structure and parameters were evaluated by X-ray diffraction (XRD) measurements using X’Pert PRO PANalytical equipment (PANalytical, Almelo, the Netherland) with a Cu Kα (λ = 1.5418 Å) radiation source in the range of 20° ≤ 2θ ≤ 80° and a step size of 0.02°/s. Structural analysis from the XRD pattern was conducted using Fullprof 2k (Institut Laue-Langevin, Grenoble, France) and VESTA software (Version 3.5.7, JP-Minerals, Nakatsugawa, Japan). Furthermore, the chemical states of La, Fe, Mn and O for each condition were investigated by X-ray photoelectron spectroscopy (XPS PHI—5400, Physical Electronics, PHI, Chicago, IL, US). The binding energy was corrected using the C1s peak at 285.0 eV. The XPS spectra were analysed using peakfit software (Version 4.11, Cranes Software International Ltd., Hyderabad, India). The room-temperature magnetic M−H hysteresis loops for calcined samples were measured by VSM (VSM 250, Xiamen Dexing Magnet Tech. Co., Ltd., Xiamen, China) with a sweeping rate of 120 Oe/s.

To form the ceramic sample, the sintering process was conducted under the following steps. First, the powder was pressed into pellets at 3 kN/inch^2^ for 3 min. Next, the pellets were sintered at 1273 K. The sintering temperature was varied for 1 and 3 h. Structural analysis was performed by XRD using the X’Pert PRO PANalytical equipment with a Cu Kα (λ = 1.5418 Å) radiation source in the range of 20° ≤ 2θ ≤ 80° and a step size of 0.02°/s. The XRD results were refined using Fullprof 2k and VESTA software. The room-temperature Raman scattering spectra were obtained using a Thermo Scientific DXR2 Raman microscope (Thermo Fisher Scientific Inc., Waltham, MA, US) with a 532-nm laser source in the range of 100–3500 cm^−1^. The electrical conductivity was investigated using an IS instrument (RLC meter, FLUKE-PM 6303, Fluke Manufacturing Company, Inc., Everett, WA, US) in the temperature range of 300–400 K and a frequency of 10 kHz.

## 3. Results and Discussion

### 3.1. Effect of Calcination Temperature on Structural and Magnetic Properties

**Figure 1:**
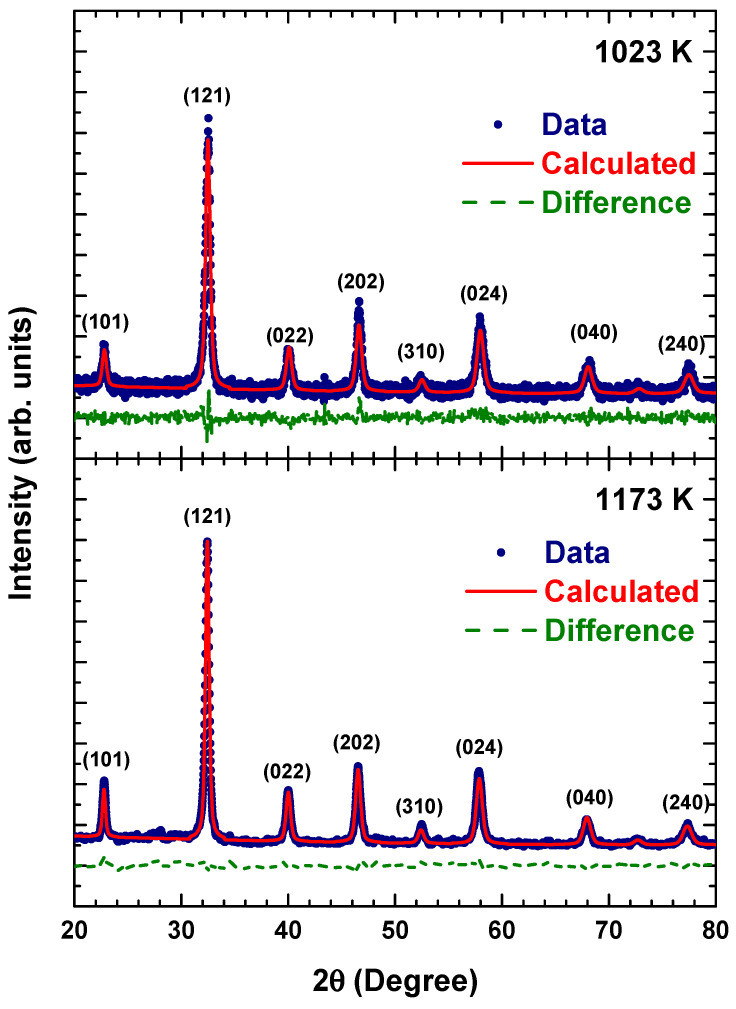
XRD patterns of La_2_FeMnO_6_ at calcination temperatures of 1023 K and 1173 K.

Figure 1 shows the XRD patterns of La_2_FeMnO_6_ calcined at 1023 K and 1173 K. For these samples, a series indexed of diffraction peaks at (101), (121), (022), (202), (310), (024), (040) and (240) indicate that the samples are crystallised in orthorhombic Pnma (62) symmetry. No additional diffraction peaks were observed above the background level, indicating a high phase purity for all samples. This result is consistent with that of previous reports for La_2_FeMnO_6_ [8,9].

The structural parameters obtained from Rietveld refinement are listed in Table 1. The sample calcined at 1173 K exhibited a larger lattice constant, volume, bond angle and crystallite size than the sample calcined at 1023 K. This is because an increase in calcination temperature promotes the formation of pure units of crystals and a high degree of crystallinity [10]. Conversely, the sample calcined at 1023 K exhibited a larger tilt angle and orthorhombic distortion than that of the sample calcined at 1173 K, indicating that the sample calcined at 1023 K created a higher lattice distortion from the orthorhombic system.

**Figure 2:**
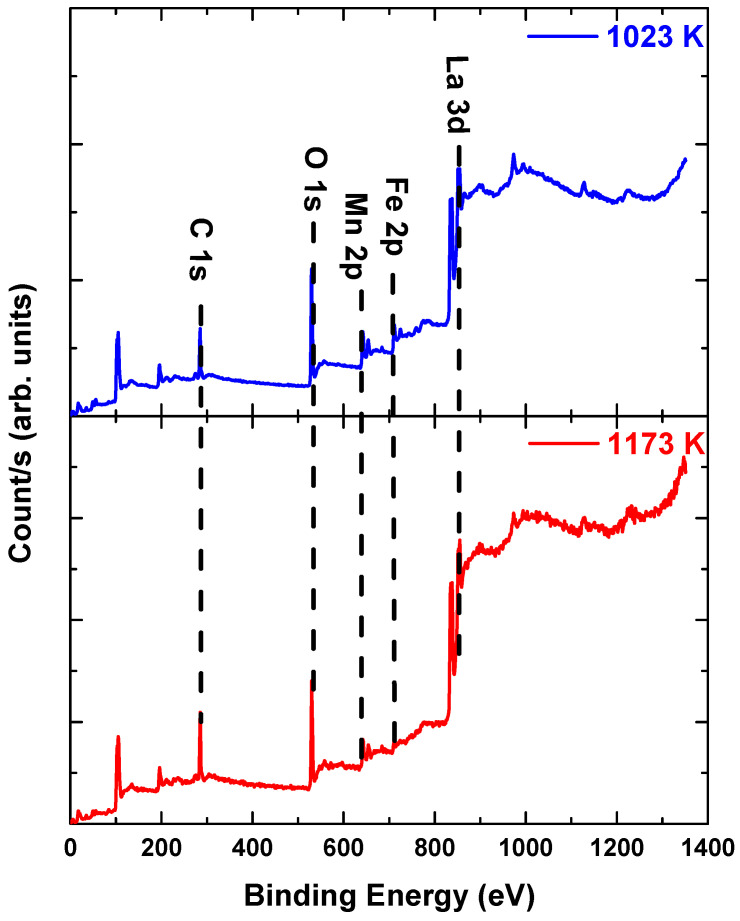
XPS spectra of La_2_FeMnO_6_ calcined at 1023 K and 1173 K.

**Figure 3:**
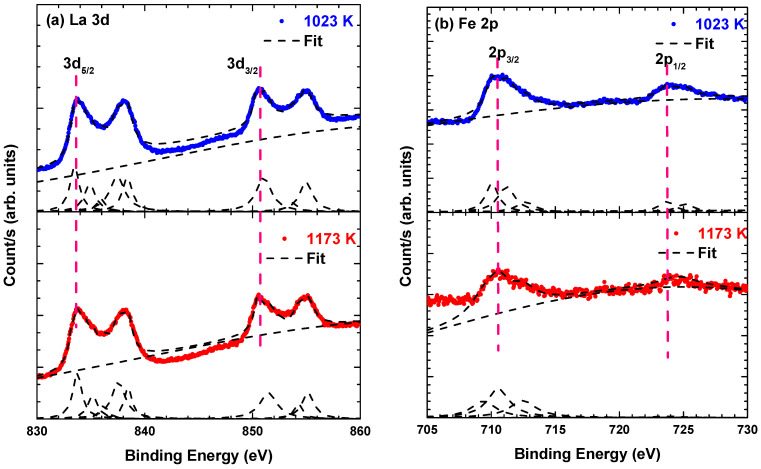

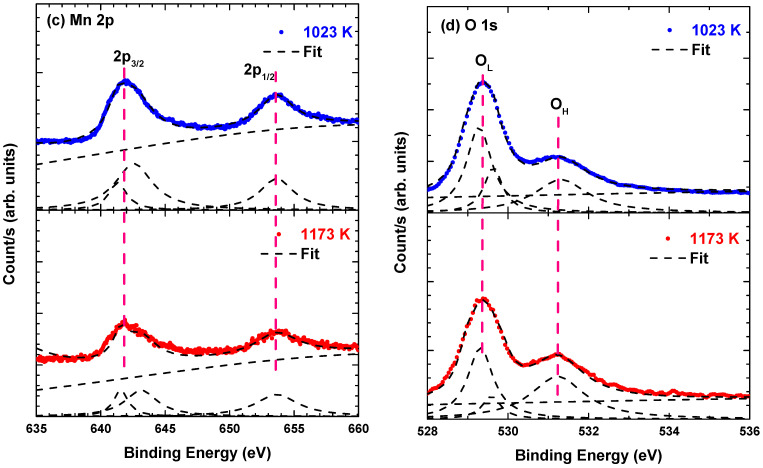
La 3d, Fe 2p, Mn 2p and O 1s spectra for La_2_FeMnO_6_ at different calcination temperatures. The blue and red dots describe the experimental data for the samples calcined at 1023 K and 1173 K, respectively. The black dashed line describes the fitting results.

Figure 2 shows the XPS spectra of La_2_FeMnO_6_ calcined at different temperatures. The spectra show the presence of La 3d, Fe 2p, Mn 2p and O 1s. The entire spectra of each sample showed no additional state or phase. The fitting results for the oxidation states for each sample are shown in Figure 3a–d. All states of La_2_FeMnO_6_ at different calcination temperatures agree well with previous reports [3,11,12,13,14,15].

Figure 3a shows the main peaks of La 3*d*_5/2_ and La 3*d*_3/2_, which show a typical La^3+^ state. The binding energies of 851.2–852.9 eV and 834.9–836.0 eV are related to the La 3d_3/2_ and La 3d_5/2_ states, respectively. Another binding energy besides that of the La 3d_3/2_ and La 3d_5/2_ states was observed. This split is associated with the shake-up state of La 3d caused by the excitation of an electron from the O 2p valence band into the unoccupied La 4f orbital [13]. The La 3d_3/2_−La 3d_5/2_ split is in the range of 16.6–16.9 eV, indicating that the La ions are in the La^3+^ state.

Figure 3b shows the Fe 2p states consisting of five components for each sample. The details of the Fe 2p states of each sample are listed in Table 2. The peak positions of Fe 2p_3/2_ and Fe 2p_1/2_ are 710.0–710.6 eV and 723.7–723.8 eV, respectively, which are attributed to the Fe^3+^ state. Other Fe states (Fe^2+^ and Fe^4+^) were observed, which are attributed to the generation of oxygen and La vacancies, respectively, as confirmed by the O 1s and La 3d spectra [14,15].

Figure 3c depicts the binding energy of Mn 2p states. The Mn 2p spectra showed two peaks at 641.2–641.4 eV and 653.5–653.7 eV, corresponding to the 2p_3/2_ and 2p_1/2_ states respectively. The spin–orbit energy spacing is approximately 12.1–12.3 eV, which is typical for the Mn^3+^ state [16]. The binding energy of La 3d and Fe 2p were shifted towards higher positions as the calcination temperature increased, suggesting an increase in La and Fe oxidising capability [13,14]. Conversely, as shown in Table 2, the Mn 2p binding energy was shifted towards a lower position with increasing calcination temperature, indicating a decrease in Mn oxidation state [13].

Figure 3d shows the XPS spectra of O 1s states for all samples. The lattice oxygen (OL) is reflected in the binding energy ranging from 529.3 to 529.6 eV, which is related to La–O and Fe/Mn–O binding. The peaks at 529.8–530.0 eV and 531.3–531.7 eV are attributed to oxygen vacancy (OV) and surface adsorbed oxygen (OH), respectively. As shown in Table 2, the peak position shifted slightly towards a higher binding energy with an increase in calcination temperature, indicating a decrease in O ions in La_2_FeMnO_6_. In other words, electrons were transferred from O to La and Fe states [17].

The surface atomic composition (%) of La_2_FeMnO_6_ at different calcination temperatures and the corresponding atomic and ionic ratios obtained by XPS are listed in Table 3. The sample calcined at 1023 K exhibited the highest Fe/La and Mn/La ratios, indicating more La vacancies in the lattice. This induced the dominance of Fe^4+^−Mn^3+^ ions, resulting in oxygen adsorption by Fe/Mn ions and La vacancies due to charge compensation [13]. The OH/O ratio decreased with increasing calcination temperature, which is in agreement with the XRD results (Table 1). With a decrease in the crystallite size, the adsorption sites for oxygen decreased.

**Figure 4:**
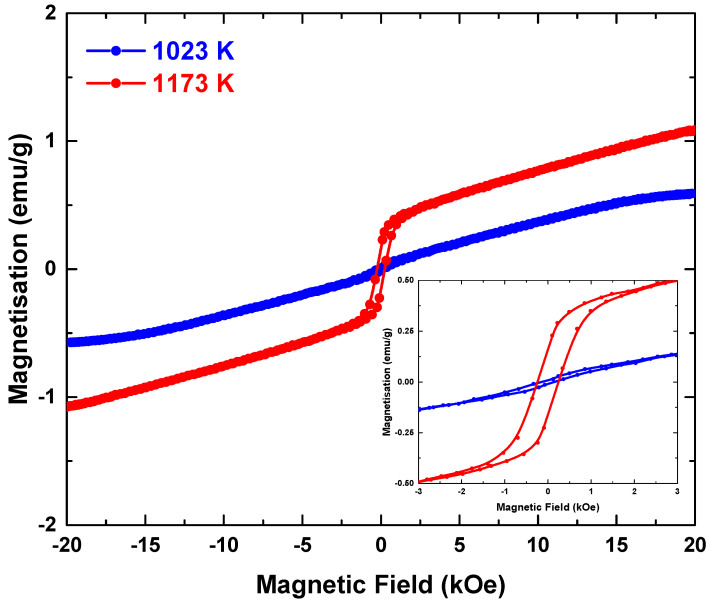
Room-temperature magnetic hysteresis of La_2_FeMnO_6_ calcined at 1023 K and 1173 K. The inset shows the hysteresis curve in the magnetic field range of −3 to 3 kOe.

Figure 4 shows the hysteresis curve of La_2_FeMnO_6_ calcined at 1023 K and 1173 K, and the inset shows the hysteresis curve in the magnetic field until 3 kOe. The magnetic parameters are listed in Table 4. A significant difference was observed as the calcination treatment conditions varied. The magnetic parameters, including the saturation magnetisation (Ms), remnant magnetisation (Mr) and coercivity (Hc), increased with calcination temperature.

Based on the XPS results, the magnetic hysteresis data were evaluated as a superposition of AFM–FIM–FM signals. The AFM part is attributed to the Fe^3+^−O–Fe^3+^ or Mn^3+^−O–Mn^3+^ clusters. Moreover, the FIM part is attributed to Fe^3+^−O–Mn^3+^, and the FM part is associated with Mn^4+^−O–Mn^3+^. Further, the <Fe/Mn–O–Fe/Mn> bond angle ranged from 161° to 162°, indicating that super-exchange interactions are favoured in the samples [9].

For the sample calcined at 1023 K, the hysteresis shows a small loop at the lower field (<1 kOe) and linear behaviour at the higher field (>1 kOe). According to the XPS results, Mn^4+^ and Fe^4+^ are dominant states in the system. However, other states, such as Fe^3+^ and Mn^3+^ are also present. The linear part was subtracted and Ms was approximately 0.59 emu/g.

For the sample calcined at 1173 K, the hysteresis curve shows a larger loop (<3 kOe) than those of the linear part at the higher field (>3 kOe). The Mn^4+^, Fe^4+^, Fe^3+^, Mn^3+^ and Fe^2+^ states were observed in the XPS analysis with Mn^3+^ and Fe^3+^ found to be dominant. Ms was approximately 1.09 emu/g.

### 3.2. Effect of Calcination and Sintering Conditions on the Structural and Electrical Properties La_2_FeMnO_6_

**Figure 5:**
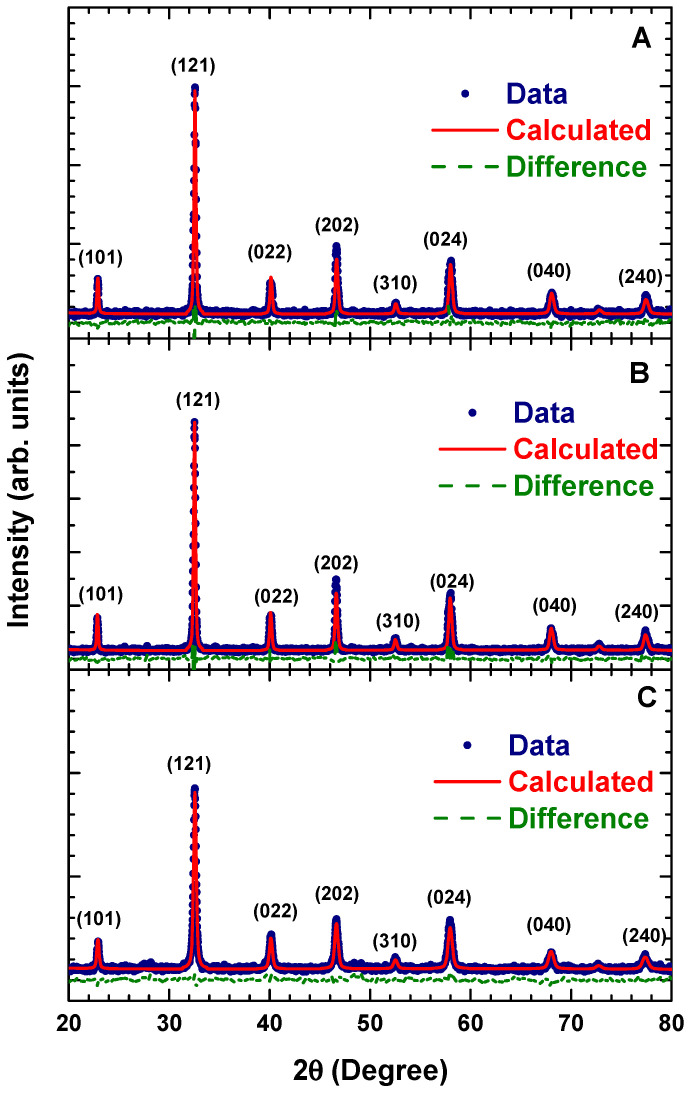
X-ray diffraction patterns of La_2_FeMnO_6_ at different calcination and sintering conditions. Conditions are labelled A (c1023 K, s1273 K, 1 h), B (c1023 K, s1273 K, 3 h) and C (c1173 K, s1273 K, 3 h).

The sintering conditions for each calcination temperature were varied. Conditions were labelled A (c1023 K, s1273 K, 1 h), B (c1023 K, s1273 K, 3 h) and C (c1173 K, s1273 K, 3 h). Figure 5 shows the XRD patterns of La_2_FeMnO_6_ at different calcination and sintering conditions.

The Miller indices of (101), (121), (022), (202), (310), (024), (040) and (240) were confirmed for each sample, indicating that all samples had crystallised in orthorhombic *Pnma* (62) symmetry. Similar to the XRD pattern of the calcined sample, no additional diffraction peaks were observed, indicating a single phase formed without any impurity phase. The Rietveld refinement results of each sample are listed in Table 5. Sample C showed a higher lattice constant, volume and bond angle, which were attributed to the better formation of crystal units [10]. However, the crystallite size showed a different trend. Sample B showed the highest crystallite size and Sample C showed the lowest crystallite size and the lowest tilt angle and orthorhombic distortion. These results confirm that varying the heat-treatment conditions can significantly affect the structural parameters, crystallinity degree and crystallite size of La_2_FeMnO_6_.

**Figure 6:**
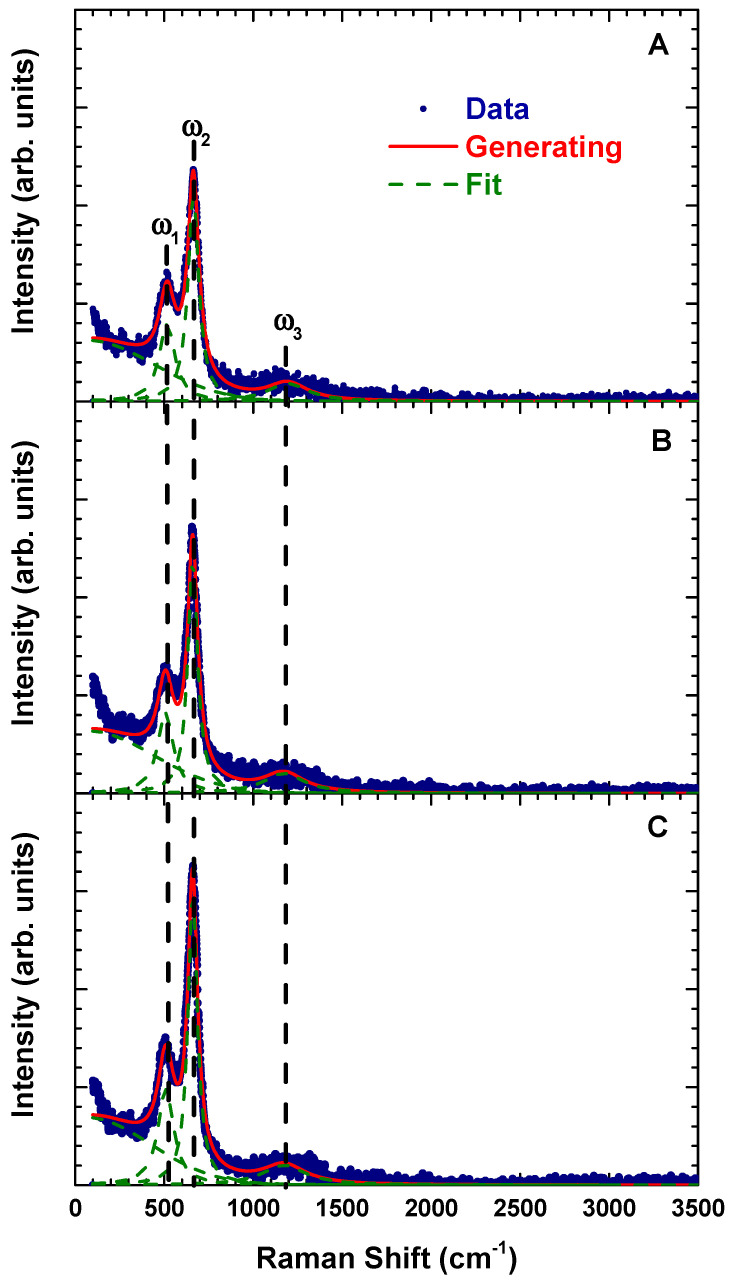
Raman scattering spectra of La_2_FeMnO_6_ at different calcination and sintering conditions. The blue dots, red line and green dashed line represent the experimental data, generated and fitting results respectively.

Figure 6 shows the Raman scattering spectra of La_2_FeMnO_6_ at different calcination and sintering conditions at room temperature. The Raman phonon modes were fitted by the standard Lorentzian technique. The two main modes ($\omega_{1}$ and $\omega_{2}$) and the high-frequency mode ($\omega_{3}$) are identified as the characteristic Raman phonon modes of La_2_FeMnO_6_ [3,4,11,12]. According to previous reports [3,4,11,12] the $\omega_{1}\;$phonon mode is related to the bending of (Fe/Mn)O_6_ octahedra with A1g symmetry, the $\omega_{2}\;$phonon mode corresponds to the stretching of (Fe/Mn)O_6_ octahedra with B1g symmetry, and the $\omega_{3}\;$phonon mode is ascribed to second-order processes which are attributed to the strong spin–lattice interactions [11,18]. No additional mode was detected in the Raman spectra indicating a high-purity La_2_FeMnO_6_ phase.

**Figure 7:**
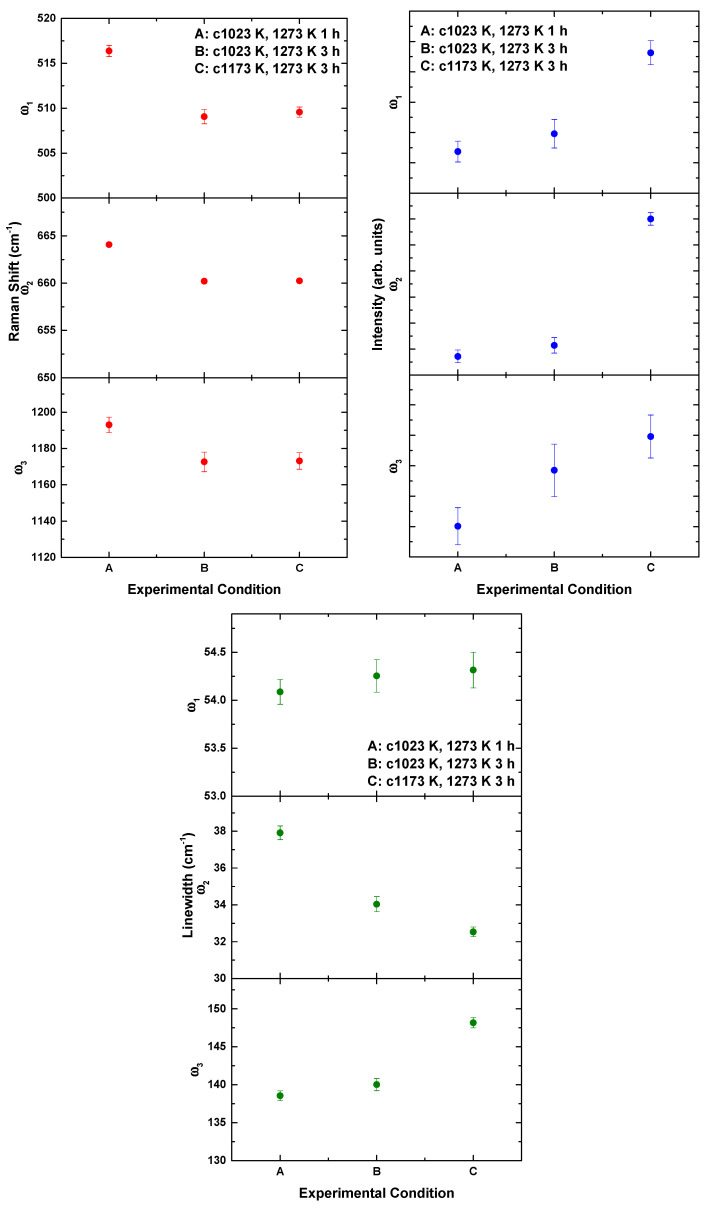
Raman phonon parameters of La_2_FeMnO_6_ at different calcination and sintering conditions.

The phonon parameters including mode position, line width and intensity are shown in Figure 7. The Raman modes were redshifted, and the intensity increased with an increase in the temperature and time of heat treatment. A different trend was observed for the linewidth. The $\omega_{1}$ and $\omega_{3}$ modes broadened, whereas $\omega_{2}$ compressed with an increase in temperature and time of heat treatment. The Raman intensity of all phonon modes was enhanced with the broadening of the line width reflecting a decrease in lattice distortion. This result is consistent with the decrease in orthorhombic distortion revealed by XRD (Table 5).

**Figure 8:**
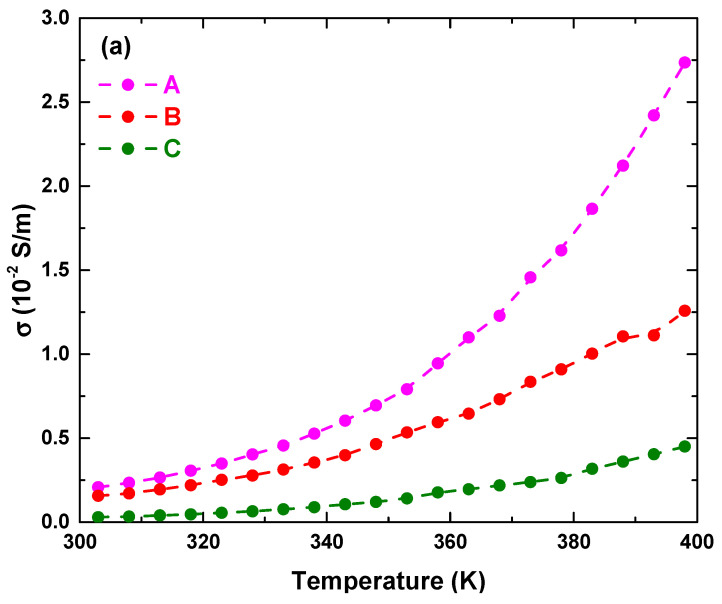

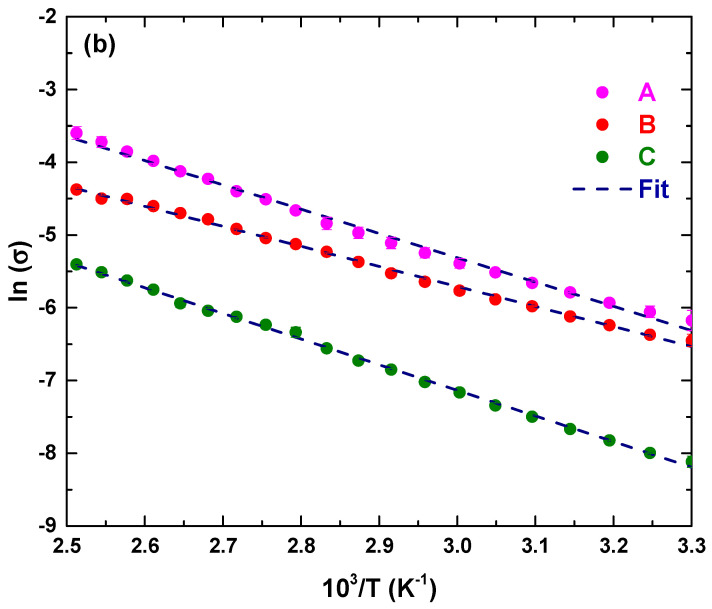
(**a**) Temperature-dependent conductivity (σ) and (**b**) fitting of σ (T) data of La_2_FeMnO_6_ at different calcination and sintering conditions using with the SPH model. E_a is approximately 0.237 ± 0.003 eV, 0.288 ± 0.005 eV and 0.304 ± 0.003 eV for samples A, B and C, respectively.

Figure 8 shows the temperature-dependent conductivity (i.e., (a) σ vs. T, and (b) ln σ vs. T-1 plots) of La_2_FeMnO_6_ under different calcination and sintering conditions. As shown in Figure 8a, σ(T) increased with an increase in temperature. Further, σ vs. T was analysed by the small polaron hopping (SPH) model, which is described as follows [3].

$$\sigma \left(T\right)=\sigma_{0}T\exp\left(\frac{E_{a}}{k_{B}T}\right)$$

with$\;\sigma_{o}$ as the pre-exponential factor, k_B_ as the Boltzmann constant (8.62 × 10^−5^ eV K^−1^) and $E_{a}$ as the activation energy required for conduction. Figure 8b shows the fitting of σ(T) data with the SPH model. There were no changes in the slope of the fitted data in each condition, indicating the existence of a single-conduction mechanism in this temperature range (300–400 K). The SPH model usually holds conduction above charge ordering in the mixed-valence manganite samples, which is a thermally activated mechanism to the weakly localised carriers [3,19]. Herein,${\;E}_{a}$ ranged from 0.20 to 1.0 eV, indicating that the conduction mechanism is dominated by p-type polaron hopping [20]. Furthermore, the lowest $E_{a}$ was obtained in Sample A, followed by Sample B and sample C. This can be explained in terms of the corresponding change in Fe/Mn–O–Fe/Mn bond angles, since the hopping conduction mechanism acts across the Fe/Mn cation sites. As revealed by XRD (Table 5), all samples showed similar Fe/Mn–O–Fe/Mn bonds indicating a similar conduction mechanism. These results showed that the heat treatment affects the conduction mechanism by lowering the activation energy, thereby increasing conductivity. A lowering of activation energy is required to improve the large electrochemical reaction in the system. This may lead to La_2_FeMnO_6_ becoming a promising candidate for use as a catalyst material on electrode surfaces in electrochemical reaction applications [20]. Consequently, this study is important for understanding fundamental physical phenomena, and for informing the development of electrodes in electrochemical systems.

## 4. Conclusions

La_2_FeMnO_6_ was synthesised via the sol–gel method. The sample was calcined at two temperatures and sintered under three conditions. Rietveld refinement analysis of XRD patterns revealed a single phase with an orthorhombic structure and Pnma (62) space group for all samples which was confirmed by XPS and Raman scattering analysis. The lattice constant, crystallite size and magnetisation parameters for the sample increased with an increase in the calcination temperature. The XPS analysis revealed changes in the Mn and Fe ionic states, and in the ionic vacancy (La and O) concentrations with calcination temperature. The electrical conductivity was based on a single-conduction mechanism dominated by p-type polaron hopping. The lowest activation energy is approximately 0.237 ± 0.003 eV at the lowest temperature and time of heat treatment. This study reveals that heating conditions significantly influence the physical properties and material quality of La_2_FeMnO_6_.

## Figures and Tables

**Table 1:** Rietveld refinement results of La_2_FeMnO_6_ calcined at different temperatures.

Parameter	Calcination Temperature (K)	
	1023	1173
Lattice parameter (Å)		
a	5.485(1)	5.507(3)
b	7.778(2)	7.805(5)
c	5.536(1)	5.523(2)
Volume unit cell (Å^3^)	236.2	237.4
Average crystallite size (nm)	213.3	325.6
Atomic position (x, y, z)		
La	(0.48, 0.25, 0)	(0.48, 0.25, 0)
Fe/Mn	(0, 0, 0)	(0, 0, 0)
O1	(0.28, 0.54, 0.23)	(0.28, 0.54, 0.23)
O2	(0.01, 0.25, 0.05)	(0.01, 0.25, 0.05)
Wickoff position		
La	4c	4c
Fe/Mn	4a	4a
O1	8d	8d
O2	4c	4c
Bond distance (Å)		
Fe/Mn–O1 (s)	1.946	1.946
Fe/Mn–O2 (m)	1.965	1.972
Fe/Mn–O1 (l)	2.019	2.022
<Fe/Mn–O>	1.977	1.980
Bond angle (°)		
Fe/Mn–O1–Fe/Mn	158.652	158.612
Fe/Mn–O2–Fe/Mn	163.486	163.577
< Fe/Mn–O–Fe/Mn>	161.069	161.095
Tilt angle (°)	11.595	11.579
Orthorhombic distortion	0.1392	0.1390

**Table 2:** Binding energy of the Fe, Mn, and O states of La_2_FeMnO_6_ calcined at different temperatures.

Calcination Temperature (K)	Binding Energy (eV)											
	Fe^3+^		Fe^2+^		Fe^4+^		Mn^3+^		Mn^4+^	OL	OV	OH
	2p_3/2_	2p_1/2_					2p_3/2_	2p_1/2_				
1023	710.05	723.71	-	711.19	712.68	725.13	641.31	653.65	642.57	529.3	529.8	531.3
1173	710.54	723.75	709.39	-	712.76	725.19	641.25	653.56	642.14	529.6	530.0	531.7

**Table 3:** Surface atomic composition (%) and the corresponding atomic and ionic ratios of La_2_FeMnO_6_ calcined at different temperatures.

Caption	Calcination Temperature (K)	
	1023	1173
Surface atomic composition (%)		
La 3d	10.2	9.76
Fe 2p	5.18	2.30
Mn 2p	6.27	4.34
O 1s	41.9	36.1
C 1s	36.5	47.5
Atomic ratio		
Fe/La	0.509	0.236
Mn/La	0.617	0.445
OH/O	0.651	0.558
Ionic ratio		
Mn^3+^/(Mn^3+^ + Mn^4+^)	0.475	0.609
Fe4+/(Fe^2+^ + Fe^3+^ + Fe^4+^)	0.589	0.418

**Table 4:** Magnetic parameters of La_2_FeMnO_6_ calcined at 1023 K and 1173 K.

Calcination Condition (K)	Ms (emu/g)	Mr (emu/g)	Hc (Oe)
1023	0.59	0.75 × 10^−2^	147.7
1173	1.09	0.16	239.9

**Table 5:** Rietveld refinement results of La_2_FeMnO_6_ at different calcination and sintering conditions. Conditions are labelled A (c1023 K, s1273 K, 1 h), B (c1023 K, s1273 K, 3 h) and C (c1173 K, s1273 K, 3 h).

Parameter	Condition		
	A	B	C
Lattice parameter (Å)			
a	5.499(3)	5.503(3)	5.518(2)
b	7.787(4)	7.792(4)	7.811(6)
c	5.533(3)	5.535(2)	5.531(3)
Volume unit cell (Å^3^)	236.9	237.3	238.4
Average crystallite size (nm)	664.5	736.6	393.3
Atomic position (x, y, z)			
La	(0.48, 0.25, 0)	(0.48, 0.25, 0)	(0.48, 0.25, 0)
Fe/Mn	(0, 0, 0)	(0, 0, 0)	(0, 0, 0)
O1	(0.28, 0.54, 0.23)	(0.28, 0.54, 0.23)	(0.28, 0.54, 0.23)
O2	(0.01, 0.25, 0.05)	(0.01, 0.25, 0.05)	(0.01, 0.25, 0.05)
Wickoff position			
La	4c	4c	4c
Fe/Mn	4a	4a	4a
O1	8d	8d	8d
O2	4c	4c	4c
Bond distance (Å)			
Fe/Mn—O1 (s)	1.947	1.948	1.950
Fe/Mn—O2 (m)	1.967	1.968	1.973
Fe/Mn—O1 (l)	2.022	2.023	2.026
<Fe/Mn—O>	1.978	1.980	1.983
Bond angle (°)			
Fe/Mn—O1—Fe/Mn	158.650	158.648	158.626
Fe/Mn—O2—Fe/Mn	163.512	163.516	163.565
Tilt angle (°)	14.396	11.587	11.579
Orthorhombic distortion	0.1387	0.1386	0.1384

## Data Availability

The data presented in this study are available on request from the corresponding author.
